# An in vitro comparison of cyclic fatigue resistance of ProTaper universal and GT series x files

**DOI:** 10.4317/medoral.18595

**Published:** 2013-02-05

**Authors:** Ramiro Montenegro-Santillán, Teresa Alegre-Domingo, Vicente Faus-Matoses, Vicente Faus-Llácer

**Affiliations:** 1 Master of Restorative Dentistry and Endodontics. Valencia University Medical and Dental School. Valencia; 2 Associate Professor of Restorative Dentistry and Endodontics. Valencia University Medical and Dental School. Valencia; 3 Professor and Director of the Master of Restorative Dentistry and Endodontics. Valencia University Medical and Dental School. Valencia (Spain)

## Abstract

Objective: The aim of this study was to compare the cyclic fatigue resistance of two nickel-titanium (NiTi) endodontic instruments from ProTaper and GT series X files. Study Design: Cyclic fatigue test was realized with instruments from ProTaper: F1 and F3 (Denstply Maillefer, Ballaigues, Switzerland) and GT-X: 20.06 and 30.08 (Dentsply Tulsa Dental, Tulsa, Oklahoma, United States of America). A total of 320 instruments were rotated in 4 curved artificial canals with different angles and radius of curvature. The time and cycles to failure were calculated. The data was compared using a Mann-Whitney, Kruskall-Wallis, and Kolmogorov-Smirnov tests, with a significance level of p<0.05. Results: GT-X files rotated for a significantly longer period of time before separation occurred, thus GT-X files where more resistant to the cyclic fatigue compared with ProTaper. Conclusion: GT-X files have a greater resistance to cyclic fatigue, this fact can be caused by the use of the Ni-Ti alloy “M-Wire”.

** Key words:**Endodontics, GT-X files, ProTaper files, cyclic fatigue.

## Introduction

The introduction of the Nickel-Titanium (Ni-Ti) rotary files in the field of endodontics has led to greater efficiency in the preparation of root canal by simplifying the process, because of the flexibility, higher fracture toughness and cutting efficiency (1,2), with less apical extrusion of debris (3) and allowing instrumentation of curved root canals with minimal transportation (4,5).

Despite having a greater flexibility compared with stainless steel files (1) they show cyclic fatigue fracture (6), that is described as the clinical failure caused by the continuous rotation of an instrument, in a curved space, in the absence of threading it, where it undergoes alternating cycles of pressure and tension. It is affected by the angle, the radius of curvature of the canal, and by the size and taper of the instrument, being the most important factor in the fracture of Ni-Ti instruments (4,7-9).

The Ni-Ti file shows no visible signs of permanent plastic deformation and separation occurs without warning, negating the validity of visual inspection of the files (7). The separation frequency of these instruments, compared to stainless steel files, is five to seven times higher (10).

In the recent research, no standarization model has been found for cyclic fatigue test, respecting to the angle of the canals, or the material used for manufacturing (7).

Recently, the GT series X files (Dentsply Tulsa Dental, Tulsa, Oklahoma, United States of America) have been developed, and incorporated into its structure Ni-Ti alloy “M-Wire” (12). It is obtained by cycles of heating and tempering during production, conferring the file a higher resistance to cyclic fatigue and greater resistance to fracture (13,14).

The GT Series X files have a rounded cutting edge, with a greater number of flutes in the tip, with a variable distance between them, eliminating the blockage of the tip into the canal. It has three different tapers and rotates at 300 rpm (14).

The aim of this study was to compare the cyclic fatigue of two Ni-Ti instrument files, GT Series X files with ProTaper Universal files (Dentsply Maillefer SA, Ballaigues, Switzerland).

## Material and Methods

The study was carried out at the University of Valencia, Medical and Dental School, to compare the cyclic fatigue of ProTaper files (Dentsply Maillefer SA, Ballaigues, Switzerland) versus GT-X files (Dentsply Tulsa Dental, Tulsa, OK, USA).

One hundred and sixty ProTaper Universal files and 160 GT-X files were used, and divided into four groups: group 1: 80 ProTa-per files F1 (tip 0.20 and taper 0.07%); group 2: 80 GT-X 20.06 files (tip 0.20 and taper 0.06%); group 3: 80 ProTaper files F3 (tip 0.30 and taper 0.09%); group 4: 80 GT-X 30.08 files (tip 0.30 and taper 0.08%).

All files were instrumented with an endodontic motor X-Smart (Denstply Maillefer SA, Ballaigues, Switzerland) 16:1 at 300 rpm, 20 files of each group were tested in all the four canals made of sintered stainless steel, to allow the reproduction of the test (7,13).

The four canals were 18 mm length, tip of 0.40 and 9% of taper. The diameter of the simulated canals was higher than files, allowing a free rotation: canal 1: 60º angle, radius of curvature of 8 mm, canal 2: 45º angle, radius of curvature of 8 mm, canal 3: 60º angle, radius of curvature of 5 mm, canal 4: 45º angle, radius of curvature of 5 mm (15).

A modification of the cyclic fatigue test (7) was used, in which sagital sections were created in the canals, a quarter of its size, and then mounted on an acrylic plate, obtaining a visual control of the fracture moment of the file and a bounded position in the acrylic plate.

**Figure 1:**
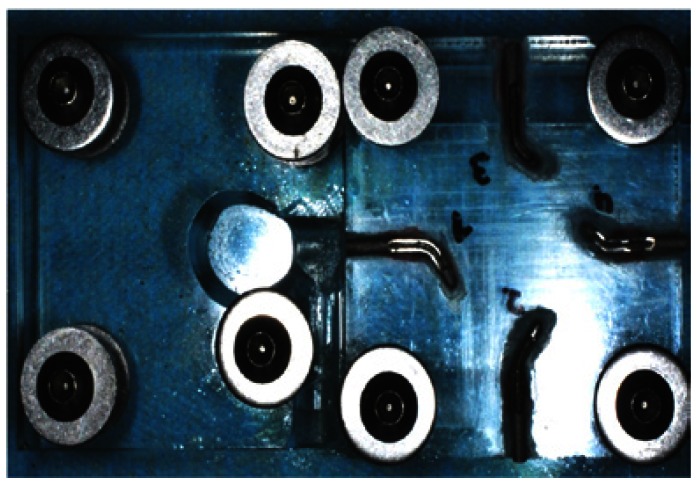
Simulated canals. Detail of the four canals with the window for irrigation, mounted on an acrylic plate fixed by screw and nut.

The handpiece was mounted, also, on an acrylic surface and clamped by a screw and nut to hold it in the same position (Fig. 1) eliminating the operator factor, which makes apical pressure in the instrumentation (7,12-17).

Instruments rotated without resistance and overheating, the simulated canals were lubricated with fluid petroleum jelly 180 (Millet-Franklin, BA, Argentina), through the window created on the surface, while each instrument rotated inside the canal (9,12,15-19).

Visual inspection of the moment of separation was performed, using SurgiTel (General Scientific Corporation, MI, USA) magnifying glasses with an increase of 2.5 x.

The instrumentation was recorded in video, and subsequently the number of rotations was calculated for each file before reaching the fracture. The number of rotations was calculated multiplying the revolutions per minute (300 rpm) for the time each file rotated, until the fracture occurred.

The data obtained was organized in four groups to compare with each other depending of the taper and the tip of the files.

The results where analyzed with SPSS 18 (SPSS Inc, IL, USA), using the Mann-Whitney, Kruskall-Wallis (for independent samples), and Kolmogorov-Smirnov test (for continuous samples), with a significance level of p<0.05.

## Results

The average number of rotations was calculated by multiplying the time in minutes per revolutions, calculating an overall average of the analyzed samples ( Table 1).

**Table 1:**
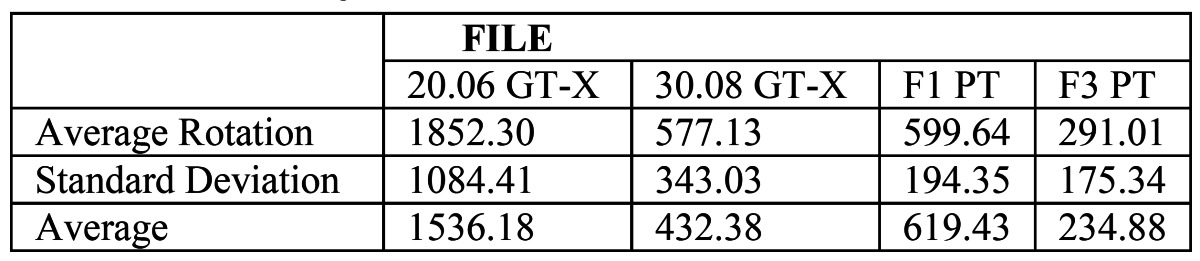
General average for the rotations of each file.

For the number of rotations in each canal, GT-X 20.06 obtained the greatest number, while ProTaper F3, showed the lowest number in all canals; a bivariant analysis was performed, and the results were statistically significant (p<0.05).

The amount of time until separation, when comparing identical file sizes of GT-X and ProTaper, was found to be greater for GT-X files, being significantly more resistant in all canals (p<0.05).

The files with 6% of taper and tip 20 (GT-X 20.06 and ProTaper F1) rotated for a longer time, resulting in a greater number of cycles, compared with the 8% taper and tip 30 (GT-X 30.08 and ProTaper F3), being the differences statistically significant (p<0.05).

**Figure 2:**
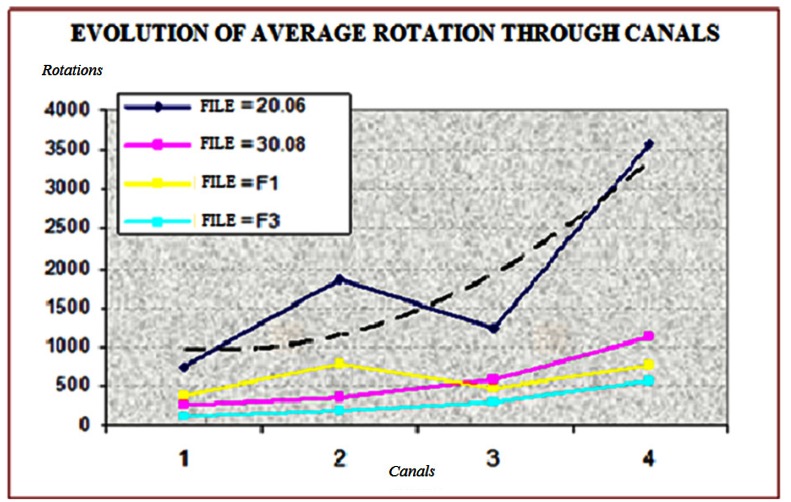
Average rotations of the files inside each simulated canal. As the canal have less curvature and angulation, the number of rotation increases.

It was shown that the canal with more complex anatomy (canal 1), generated a lower number of rotations (in the four groups compared), being the average number of the rotations in this canal much less than the average number in the canal 4 (easier canal), which generates the largest number of rotations for all files, the differences where statistically significant (p<0.05) (Fig. 2).

## Discussion

File separation is a major concern during the endodontic treatment, despite the separation may occur by multiple factors, the cyclic fatigue is one of the leading causes (4).

This study compared the cyclic fatigue of ProTaper Universal files (Maillefer SA, Ballaigues, Switzerland) with the GT Series X files (Dentsply Tulsa Dental, Tulsa, Oklahoma, USA).

Files selected were Protaper F1 and ProTaper F3 (Maillefer SA, Ballaigues, Switzerland) and GT Series X 20.06 and 30.08 (Dentsply Tulsa Dental, Tulsa, Oklahoma, USA) because those have the same tip diameter and a similar taper in the first three millimeters of each file (ProTaper F1 and F3 files have 0.01% more taper). The two most important factors when comparing different instrumentation systems are the instrument volume and taper; to eliminate possible conditioning factors of the study same files were used (13,14,20), as Al-Hadlaq et al. (22) that compared K3 20.06 with GT-X 20.06 files founding no significant differences among the two files; in this study the differences between ProTaper and GT-X files where significant.

The incorporation of Ni-Ti “M-Wire” alloy in the GT Series X files can improve the resistance to intracanal failure; in recent research, there are few articles that compare GT Series X files versus files without any modification in the fabrication process, with similarities in volume, size and taper (21). However, this study compared two different file systems, with different geome-tries, which were used for finishing the instrumentation of the root canal.

ProFile GT and ProFile GT Series X where also compared in canals with curvature of 45º and 60º, with an apical movement of 10 revolutions per minute (23). In that study, no significant differences were found. However, more recent studies (24) found differences with respect to the cyclic fatigue, which is dependent of the thermomechanical treatment applied in GT-X. These results are similar to this study, in which no apical motion was used and the free rotation of the files was allowed.

The volume of the instrument affects the results of cyclic fatigue, because as the instrument size increases, the time to reach the fracture decreases (7,10). In this study we found that ProTaper F3 was the less resistant file to cyclic fatigue due to the volume that it own.

The cyclic fatigue was also compared between GT Series X 20.04 and 20.06 with Twisted Files, EndoSequence and ProFile, with a tip diameter of 25 (19), obtaining a greater number of rotation for the GT Series X files 20.04 and 20.06. This results are attributable to the mass difference between the compared files. In the present study, the volumetric size of the files was the same.

The cyclic fatigue resistance for F1 and F3 files is similar depending of the volume, but independent of the geometry of the canal (16), being F3 the one that shows less resistance. These results are consistent with our results.

The fracture test performed in this study showed that the place of separation is located at the midpoint of the curvature. These results were previously found by other researchers (7,15).

In conclusion, GT Series X 20.06 and 30.08 files, had a greater resistance to cyclic fatigue compared to ProTaper Universal F1 and F3 files. In the “in vitro” test, this could be attributable to the manufacturing process in which files are subjected to heating and tempering.

## References

[1] Walia HM, Brantley WA, Gerstein H (1988). An initial investigation of the bending and torsional properties of Nitinol root canal files. J Endod.

[2] Kazemi RB, Stenman E, Spangberg LS (2000). A comparison of stainless steel and nickel titanium H-type instruments of identical design: torsional and bending tests. Oral Surg Oral Med Oral Pathol Oral Radiol Endod.

[3] Ferraz CC, Gomes NV, Gomes BP, Zaia AA, Teixeira FB, Souza-Filho FJ (2001). Apical extrusion of debris and irrigants using two hand and three engine-driven instrumentation techniques. Int Endod J.

[4] Sattapan B, Nervo GJ, Palamara JE, Messer HH (2000). Defects in rotary nickel-titanium files after clinical use. J Endod.

[5] Glossen CR, Haller RH, Dove SB, del Rio CE (1995 ). A comparison of root canal preparations using Ni-Ti hand, Ni-Ti engine-driven, and K-Flex endodontic instruments. J Endod.

[6] Yared G (2004). In vitro study of the torsional properties of new and used ProFile nickel titanium rotary files. J Endod.

[7] Pruett JP, Clement DJ, Carnes DL (1997). Cyclic fatigue testing of nickel-titanium endodontic instruments. J Endod.

[8] Iqbal MK, Kohli MR, Kim JS (2006). A retrospective clinical study of incidence of root canal instrument separation in an endodontics graduate program: a PennEndo database study. J Endod.

[9] Grande NM, Plotino G, Pecci R, Bedini R, Malagnino VA, Somma F (2006). Cyclic fatigue resistance and three-dimensional analysis of instruments from two nickel-titanium rotary systems. Int Endod J.

[10] Alapati SB, Brantley WA, Svec TA, Powers JM, Nusstein JM, Daehn GS (2005). SEM observations of nickel-titanium rotary endodontic instruments that fractured during clinical use. J Endod.

[11] Parashos P, Gordon I, Messer HH (2004). Factors influencing defects of rotary nickel-titanium endodontic instruments after clinical use. J Endod.

[12] Kell T, Azarpazhooh A, Peters OA, El-Mowafy O, Tompson B, Basrani B (2009). Torsional profiles of new and used 20/.06 GT series X and GT rotary endodontic instruments. J Endod.

[13] Haïkel Y, Serfaty R, Bateman G, Senger B, Allemann C (1999). Dynamic and cyclic fatigue of engine-driven rotary nickel-titanium endodontic instruments. J Endod.

[14] Buchanan LS (2008). The new GT Series X rotary shaping system: objectives and technique principles. Dent Today.

[15] Ruddle CD (2001). The Protaper technique: endodontics made easier. Dent Today.

[16] Kramkowski TR, Bahcall J (2009). An in vitro comparison of torsional stress and cyclic fatigue resistance of ProFile GT and ProFile GT Series X rotary nickel-titanium files. J Endod.

[17] Ounsi HF, Salameh Z, Al-Shalan T, Ferrari M, Grandini S, Pashley DH (2007). Effect of clinical use on the cyclic fatigue resistance of ProTaper nickel-titanium rotary instruments. J Endod.

[18] Kitchens GG, Liewehr FR, Moon PC (2007). The effect of operational speed on the fracture of nickel-titanium rotary instruments. J Endod.

[19] Lopes HP, Moreira EJ, Elias CN, de Almeida RA, Neves MS (2007). Cyclic fatigue of ProTaper instruments. J Endod.

[20] Inan U, Aydin C, Tunca YM (2007). Cyclic fatigue of ProTaper rotary nickel-titanium instruments in artificial canals with 2 different radii of curvature. Oral Surg Oral Med Oral Pathol Oral Radiol Endod.

[21] Larsen CM, Watanabe I, Glickman GN, He J (2009). Cyclic fatigue analysis of new generation of nickel titanium rotary instruments. J Endod.

[22] Al-Hadlaq SM, AlJarbou FA, AlThumairy RI (2010). Evaluation of cyclic flexural fatigue of m-wire nickel-titanium rotary instruments. J Endod.

[23] Gambarini G, Grande NM, Plotino G, Somma F, Garala M, De Luca M (2008). Fatigue resistance of engine-driven rotary nickel-titanium instruments produced by new manufacturing methods. J Endod.

[24] da Cunha Peixoto IF, Pereira ES, da Silva JG, Viana AC, Buono VT, Bahia MG (2010). Flexural fatigue and torsional resistance of ProFile GT and ProFile GT Series X Instruments. J Endod.

